# The neural substrate of navigation using hydrostatic cues in goldfish

**DOI:** 10.1098/rsos.241869

**Published:** 2025-02-05

**Authors:** Shachar Givon, Renana Altsuler-Nagar, Ronen Segev

**Affiliations:** ^1^ Department of Life Sciences, Ben-Gurion University of the Negev, Beer-Sheva, Israel; ^2^ Department of Biomdeical Engineering, Ben-Gurion University of the Negev, Beer-Sheva, Israel

**Keywords:** teleost, depth, sensing, spatial, cognition, telencephalon, navigation

## Abstract

Hydrostatic pressure is a global sensory cue exploited by fish to navigate in the vertical dimension. Unlike other navigational cues in the horizontal plane that usually require learning and memory to determine location, hydrostatic pressure signals the absolute position along the vertical axis. Recently, it was shown that fish can use hydrostatic signals to navigate. It remains unclear, however, which brain regions are involved in processing this signal. Here, we tested whether the dorsomedial and lateral parts of the pallium, two regions that were found to be critical in horizontal navigation, are also critical for hydrostatic cue detection in goldfish. The results show that lesions to both these regions cause fish performance to deteriorate to chance values, indicating that both regions play an important role in processing hydrostatic pressure cues. These findings thus contribute to the rapidly growing body of knowledge on teleost navigation in space.

## 1. Introduction

Navigation is critical for the survival of almost all animals, including fish. Studies have shown that fish can navigate using both allocentric and egocentric cues [1–4] as well as estimate distance [5]. In addition, fish can use beaconing in [6] and out of their natural environment [7], and have preferred locations in the form of a home base [8]. However, while most land animals navigate in a primarily two-dimensional world, the aquatic environment is three-dimensional by nature. This additional dimension is not solely geometric since movement along the vertical axis results in differences in hydrostatic pressure. Fish can use this global cue for precise localization in the water column [9].

A recent study showed that Mexican tetra fish can use pressure cues to orient themselves towards food targets [9]. The fish were tested in a set-up where the depth of a food item was kept constant while all other cues were altered and unrelated to the location of the food item. Although the results pointed to the existence of pressure perception in teleost fish, it remains unclear where in the fish brain this information is processed for the purposes of navigation.

Studies in the last few years have shown that the fish telencephalon is a critical component of fish navigation [10,11]. Specifically, electrophysiological recordings from the lateral and central telencephalon in behaving fish have demonstrated the existence of cells encoding different aspects of fish location and movements [12–15]. We will use the terms ‘lateral pallium (LP)’ and ‘medial pallium (MP)’ solely to refer to the lateral and medial parts within the mature teleost pallium, in a purely topographical sense. These terms are not intended to reference the developmental origin as used in the nomenclature by Puelles *et al*. [10], Mueller *et al*. [16] and more. Lesion studies have attempted to determine which areas of the telencephalon are crucial for spatial cognition. The results vary across research groups and experimental set-ups since some studies have reported that lesions to the lateral part of the pallium (LP) result in impairments to spatial memory [2,11,17], while others have singled out the medial part of the pallium (MP) as the dominant region [16,18,19]. Comprehensive studies using a combination of two-dimensional navigational set-ups such as the plus maze and the breadboard have arrived at the conclusion that lesions to either region are detrimental to spatial cognitive memory [16].

Here we combined a behavioural task with a lesion study to determine whether cognitive pressure cues are indeed coded in the telencephalon. Success in this task required the fish to use its sense of depth or hydrostatic pressure. We predicted that a lesion to the region where such information is putatively coded would lead to a deterioration in the fish’s ability to reach its target. The findings indicate that both LP and MP are involved in this process.

## 2. Methods

### 2.1. Animals

All experiments were approved by the Ben-Gurion University of the Negev Institutional Animal Care and Use Committee and were in accordance with the government regulations of the State of Israel. The fish length was 15−18 cm from head to tail and 80−120 g body weight. A total of 38 male and female fish, ages unknown, were used in the study. The fish were kept at room temperature in a water tank and the room was illuminated with a 12/12 h day−night cycle. The fish were kept in their home water tank and for the behavioural experiments they were relocated to the experimental water tank. The fish were fed with the experimental food reward and habituated to the laboratory for at least two weeks prior to the experiments.

### 2.2. Behavioural arena

**Figure 1.**
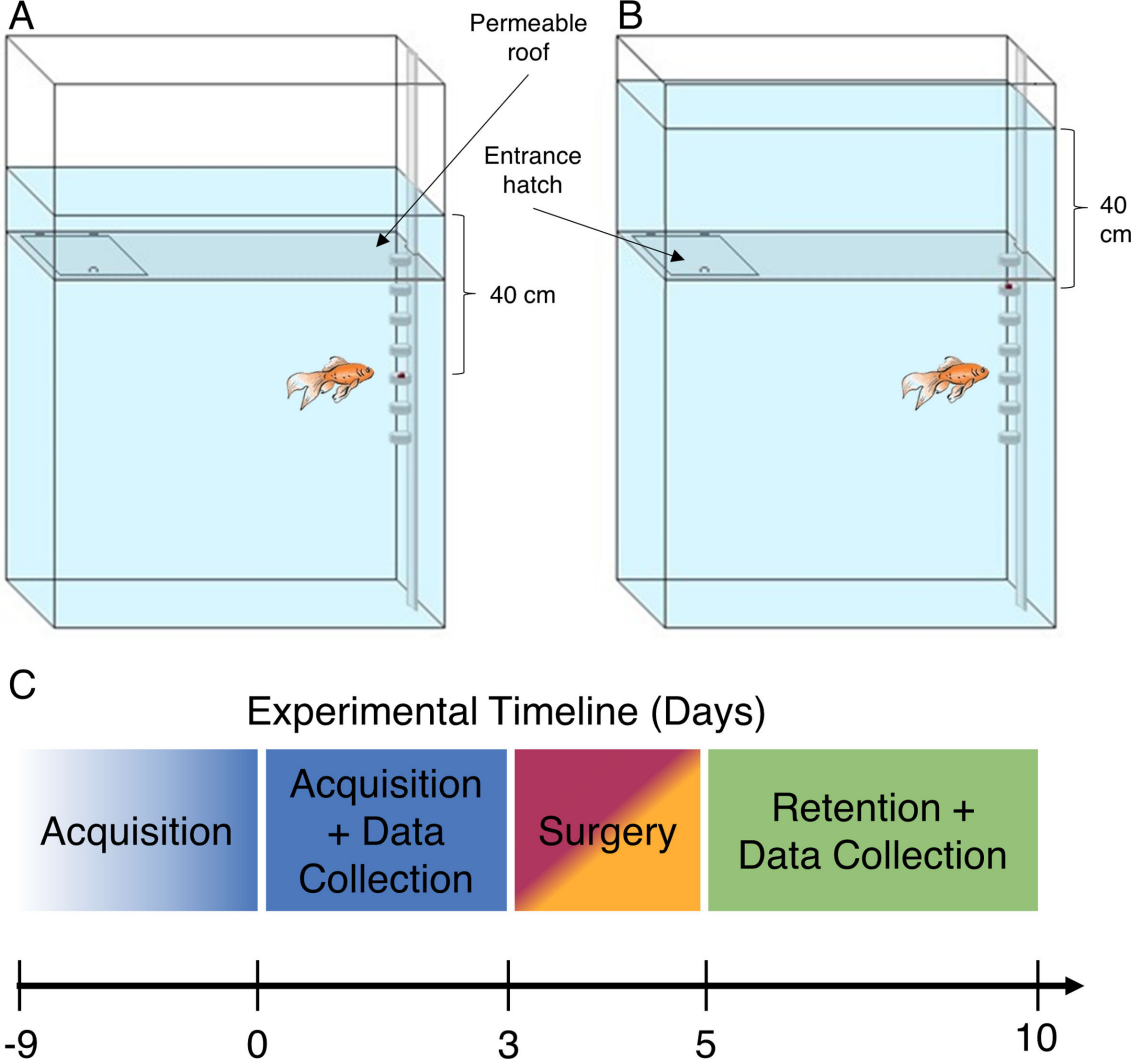
Schematic view of the experimental set-up. The experimental set-up consisted of a vertical water tank 22 cm × 75 cm × 110 cm. The roof of the arena for fish exploration was non-transparent but permeable to water added from the top. The roof was stationary, whereas the water level could be changed by adding or removing water from the top. Food was always located at a constant depth of 40 cm below the surface of the water. Fish entered through a hatch in the water-permeable roof. A. Water corresponding to the third basket from the bottom. Fish reaching the target. B. Water level corresponding to the sixth basket. Fish reaching an incorrect basket, error of −15 cm.

The behavioural arena consisted of a vertical water tank (22 cm × 75 cm × 110 cm) with a water-permeable but opaque roof situated 70 cm above the tank bottom (figure 1). The roof was designed to make it possible to insert the fish, the feeding column and provide an unobstructed water flow. The water level was set between 70 and 100 cm as a function of the experimental conditions. The feeding column was positioned at one edge of the tank and had seven feeding baskets at heights of 30–60 cm above the water tank bottom, spaced at 5 cm intervals. The fish were placed rapidly into the arena using a net that afforded little visibility to prevent the fish from orienting themselves by using the visual cue of the water level.

### 2.3. Behavioural experiment

The fish were randomly assigned to one of four experimental groups: control (*n* = 8), sham (*n* = 10), LP lesion (*n* = 10) and MP lesion (*n* = 10). After two orientation sessions of 2 days of free exploration of the vertical tank for half an hour, the fish started the training phase. In each trial, a fish was placed in the vertical water tank. The food pellet was only available in the basket 40 cm below the water’s surface, which could not be seen by the fish unless it approached the basket. The fish were then allotted 3 min to reach the basket containing the pellet. Prior to each session, the seven water levels and their order were randomly assigned using a pseudo-random number generator.

Success was defined as reaching the correct basket on the first attempt in less than 3 min. A fish was said to fail if it went to an incorrect basket within the time window. Fish that did not attempt to reach any basket within the designated time were labelled ‘no selection’. Each fish was tested 10 times during one daily session until they achieved a 50% cumulative success rate on three successive daily sessions. This criterion was based on simulations showing that the probability of achieving 50% success without a learning process, hence by random choice, is $5\;\times 10^{-5}$. Then each fish underwent surgery, was given a couple of days to rest and recover and was returned to the tank for five more days of testing.

### 2.4. Determining success criteria

Before any learning occurred, the success rate was 14.29%. After running simulations, the cumulative success criterion was chosen to be 50%. The probability of reaching a 50% success rate in three successive days when making only random choices was less than $5\times 10^{-5}$. Hence, fish that met the criteria for success most likely did not do so randomly.

### 2.5. Surgery

Surgery followed the procedure in [16]. Prior to surgery, the fish were anaesthetized in water containing 200 ml MS-222 (Cat A-5040, Sigma-Aldrich, St Louis, MO, USA) per litre. Then, the fish were wrapped in a wet towel, preventing drying of the skin, and contained in a respiratory system containing MS-222. Skin was removed above the skull with the use of a scalpel, Lidocaine (L-7757, Sigma-Aldrich, St. Louis, MO, USA) and povidone-iodine were applied for local anaesthesia and disinfection, respectively.

Screws were mounted into the skull around the surgical area for the later applied dental cement to have a better hold. There, above the telencephalon, a wider aperture was made and the removed piece of skull was immersed in PBS for later reattachment. The lesioned tissue, LP or MP, was separated and suctioned out using a set of different width needles, 0.6 mm × 30 mm and 0.5 mm × 16 mm (Suzhou Texnet Co., China) and a vacuum pump (ME 2C, Vacuubrand, Germany). The removed piece of skull was then reattached using dental cement (GC Fuji PLUS, 001409, GC Corporation, Japan). The fish were reanimated with the use of fresh water in the respiratory system instead of the MS-222 mixture and returned to their home tank for monitoring [16,20].

Fish in the sham group had their skull piece reattached shortly after removal without any further intervention, whereas the fish in the control group did not undergo any surgical treatment.

### 2.6. Histology

Histology followed the procedure in [16]. At the end of the behavioural experiment, all fish were sacrificed with an overdose of MS-222 and the brains were removed from the fish. Then, the brains of the fish were placed for 24 h in 4% paraformaldehyde for fixation (Electron Microscopy Sciences, CAS #30525-89-4). This was followed by 24 h rinsing in a glucose solution (40%). The brains were then embedded in an Optimal Cutting Temperature Compound (Scigen Scientific, Gardena, CA, USA) and were frozen in liquid nitrogen. Samples were sliced using a Cryostat CM3050S (Leica Biosystems, USA) at 40 μm thickness. Finally, slices were scanned using a Panoramic Scanner.

### 2.7. Statistical analysis

To evaluate differences between the groups and between pre/post-surgical data, two-way ANOVA tests were used. All analyses and statistics were done using MATLAB programs.

## 3. Results

To test whether the lateral and medial parts of the pallium were involved in the processing of hydrostatic sensory signals, we used a set-up where the fish had to use hydrostatic cues to find a food reward while all other sensory cues remained constant (figure 1A). The behavioural arena consisted of a feeding column at one edge of the tank with seven feeding baskets at heights of 30−60 cm above the water tank bottom (at 5 cm intervals). The roof of the behavioural arena permitted the insertion of the fish and allowed water from above to exert pressure on the bottom. The roof was opaque such that the fish experienced a visually constant environment. By adjusting the water from above the roof, it was possible to place a food reward in different baskets while keeping the reward at a constant depth of 40 cm below the water level. A total of 38 fish were divided randomly into four groups: control (*n* = 8), sham (*n* = 10), LP lesion (*n* = 10) and MP lesion (*n* = 10).

### 3.1. Lesions to MP and LP had an effect on retention of depth cues

**Figure 2.**
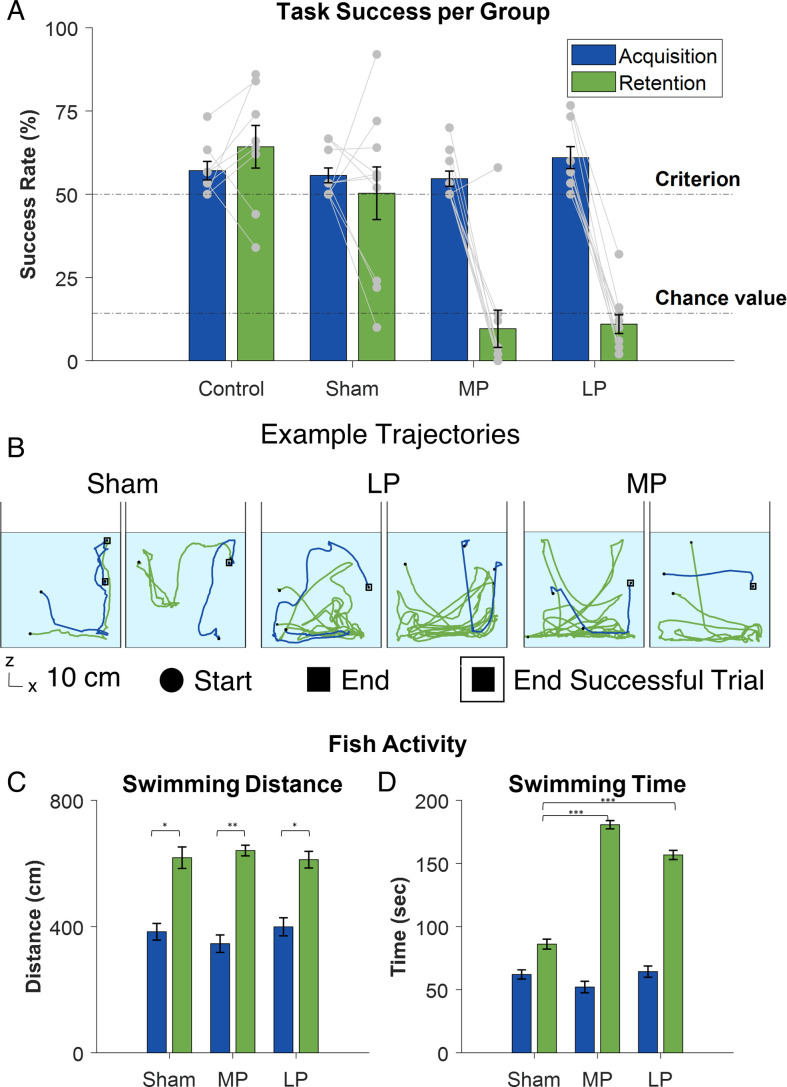
Performance across the different groups of fish during the training and testing phases. A. The success of each group before and after surgery. The sham and control groups exhibited a high success rate whereas LP and MP declined below chance. B. Examples of swimming trajectories in six fish, two from each group, before and after surgery. The framed square indicates that the fish reached the target. C. Swimming distance before and after surgery. All groups exhibited an increase. D. Swimming time until the end of the trial for each group before and after surgery. The LP and MP groups took significantly longer to finish the trials in the retention phase than the sham group.

Figure 2A presents the success rate for each group before and after the surgical procedure. The data consist of the last 3 days before surgery (30 trials per fish) and the 5 days after surgery (a total of 50 trials per fish). At the end of the training phase, i.e. before the surgical intervention, no significant differences were observed between the surgical groups and the control (two-way ANOVA, $p_{\left(control-sham\right)}$ = 0.98, $p_{\left(control-MP\right)}$ = 0.93, $p_{\left(control-LP\right)}$ = 0.78).

The pre- and post-surgery comparison revealed no significant difference between the control and sham groups (two-way ANOVA, $p_{\left(control\right)}$ = 0. 2, $p_{\left(sham\right)}$ = 0.37). By contrast, both the LP and the MP groups showed a significant decline in success rates in comparison with their success rate before surgery (two-way ANOVA, $p_{\left(MP\right)}$ = 2 $\times 10^{-16}$, $p_{\left(LP\right)}$ = 1 $\times 10^{-19}$). The fish did not exhibit signs of improvement the further the experiment extended.

The overall activity of the fish after surgery was assessed in terms of the distance the fish travelled in each trial and the trial duration. Figure 2B depicts examples of the swimming trajectories of fish before surgery (blue lines) and after (green lines). Figure 2C compares the mean swimming distance for each group before and after surgery. In all groups, there was a significant increase in swimming distance after surgery (two-way ANOVA, $p_{\left(sham\right)}$ = 6 $\times 10^{-7}$, $p_{\left(MP\right)}$ = 1 $\times 10^{-19}$, $p_{\left(LP\right)}$ = 2$\;\times 10^{-7}$). The differences between groups were not significant. A comparison of swimming times between the sham group and the MP and LP lesioned fish was not significant either before or after surgery (two-way ANOVA, $p_{pre}\left(sham-LP\right)$ = 0.9, $p_{pre}\left(sham-MP\right)$ = 0.54, $p_{post}\left(sham-LP\right)$ = 0.98, $p_{post}\left(sham-MP\right)$ = 0.82).

By contrast, there were significant differences between the surgical groups concerning trial duration. As seen in figure 2D, there was no significant difference between groups pre-surgery (two-way ANOVA, $p_{pre}\left(sham-LP\right)$ = 0.91, $p_{pre}\left(sham-MP\right)$ = 0.15) but a significant increase post-surgery (two-way ANOVA, $p_{\left(sham\right)}$ = 2$\times 10^{-5}$, $p_{\left(MP\right)}$ = 3 $\times 10^{-75}$, $p_{\left(LP\right)}$ = 2 $\times 10^{-42}$) . However, the increase in swimming time after surgery was significantly higher for the MP and LP lesioned groups than for the sham group (two-way ANOVA, $p_{post}\left(sham-LP\right)$ = 0, $p_{post}\left(sham-MP\right)$ = 2 $\times 10^{-15}$). These results show that all the fish remained active after surgery and that there was no impairment to their motor ability to complete the task.

### 3.2. Fish with lesions fail to complete the task

**Figure 3.**
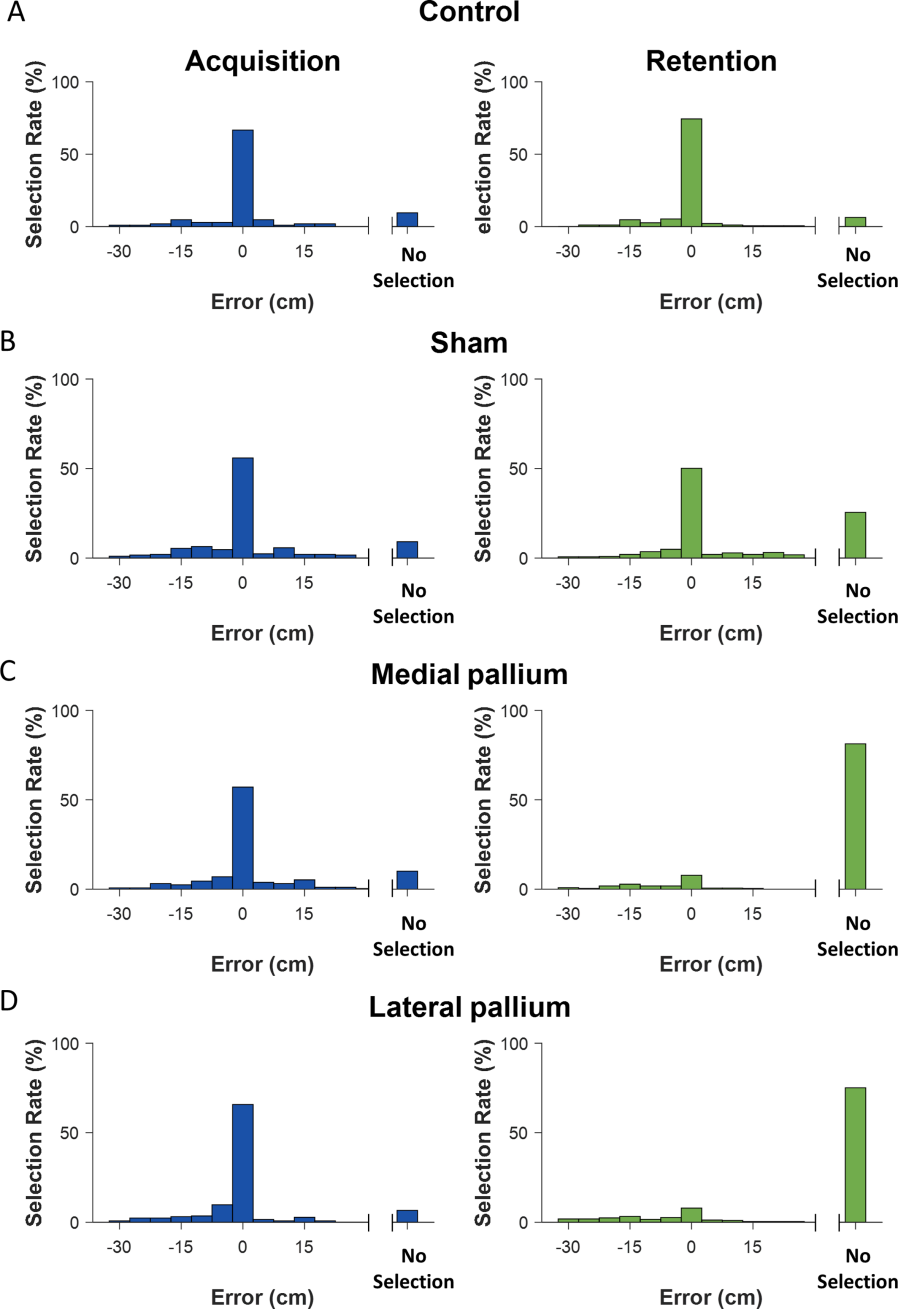
Vertical errors in search depth. Vertical errors for the acquisition and retention phases. An error of zero indicates the correct search location. ‘No selection’ indicates that the fish did not search in any of the baskets during the allotted time. A. control group. B. sham group. C. MP group. D. LP group.

Figure 3 presents histograms of the distances between the fish selections and the true target location. For the control group, there was no significant difference pre- versus post-surgery (two-way ANOVA, *p* = 0.9), in that the fish looked for the food in the correct basket and did not search for any basket only a small percentage of the time. The mean distance from the target during the test phase was −1.15 cm with a standard error of 0.7 cm.

There was a significant difference in the sham group between selection behaviour before and after surgery (two-way ANOVA , *p* = 0.03) with the mean changing from a negative −1.1 cm for the target with a standard error of 0.55 cm to a positive value of 0.4 cm mean with a 0.4 cm standard error.

The LP group presented a significant difference between pre- versus post-surgery (two-way ANOVA, *p* = 3$\;\times \;10^{-7}$). This was most pronounced when the fish did not attempt to search in any of the baskets. When the fish searched for food, the mean distance error from the target after surgery was −7 cm with a standard error of 1.14 cm, and hence was much higher than before surgery when the mean was −2 cm with a 0.4 cm standard error. Overall, when attempting food search, the fish had only a 30% success rate.

In the MP group, like the LP group, the fish ignored the baskets a large percentage of the time after surgery. There was a significant difference in the distance travelled in relation to pre-surgical performance (two-way ANOVA, *p* = $1\;\times \;10^{-8}$). The mean before surgery was −0.3 cm with a 0.5 cm standard error, whereas after surgery the mean dropped to −6.6 cm with a 1 cm standard error. When attempting food search, they had a 41% success rate.

Finally, it is important to mention that for LP lesion groups, when the animal selected, the selection was 70% wrong. For the MP lesion group, 59% of the selections are not the correct selection. Thus, when there are selections, they are mostly wrong selections in the two lesion groups.

### 3.3. Relationship between lesion size and post-lesion success rate

We controlled for a possible relationship between hydrostatic pressure, memory performance and fractional lesion size in the specific brain regions. The lesions had a significant effect, as indicated in the comparisons of the sham and control groups to the surgery groups. However, we suspected that there may have been a residual dependence on the size of the lesion.

**Figure 4.**
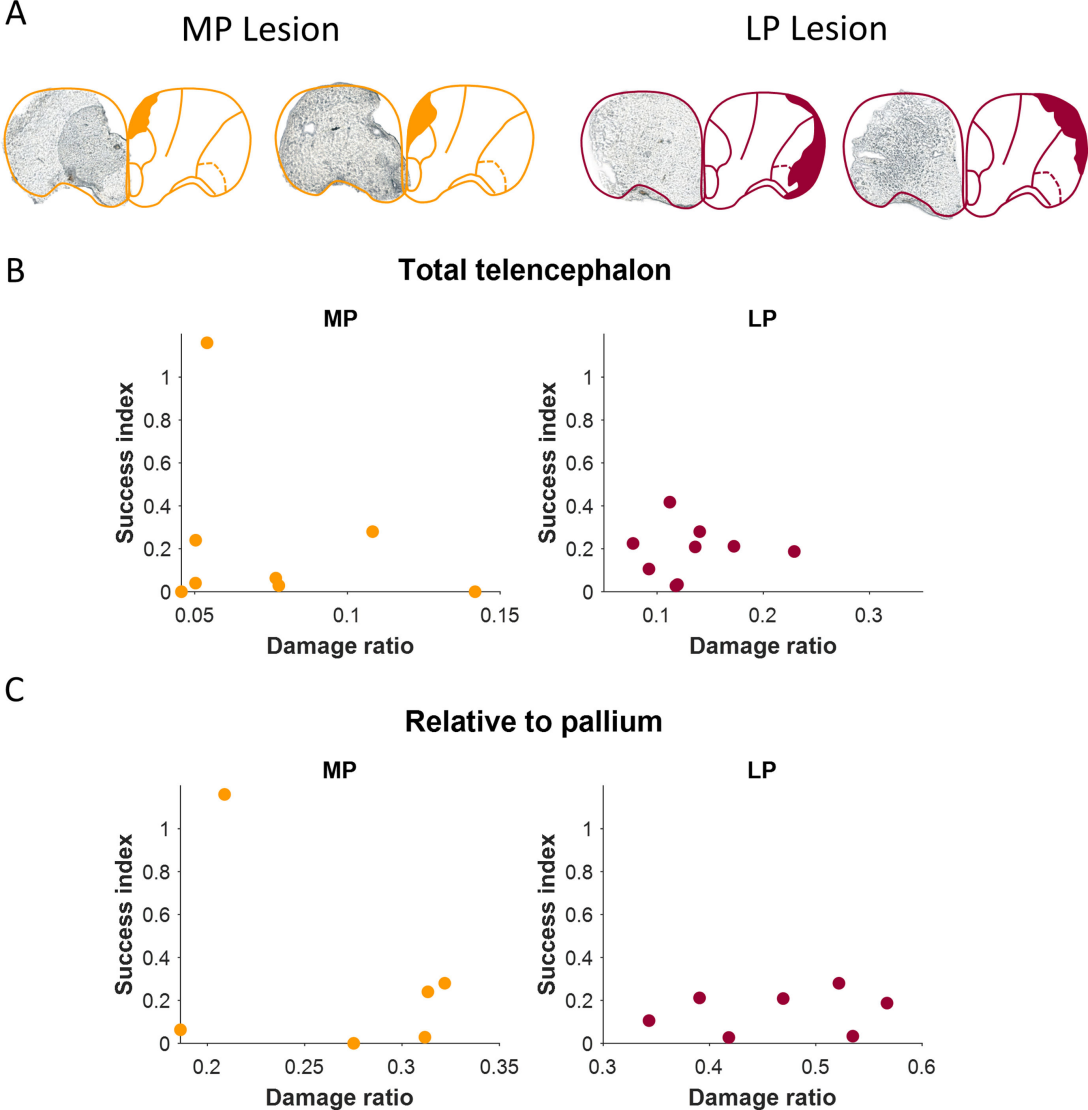
Relationship between lesion size and success. A. Examples of lesions to the MP (yellow) and LP (red). The mask is based on Northcutt [3] B. Success expressed as the ratio of pre- to post-surgery success, as a function of total telencephalon damage, for each lesion type. C. Success, as a function of pallium damage, for each lesion type.

Thus, we measured the lesion size in the medial and lateral regions for each fish. Figure 4A shows examples of brain slices of lesioned fish. Although all lesions were performed by the same surgeon and in the same manner, the lesions indeed varied in location and size. Figure 4B,C shows each fish’s performance in behavioural experiments and the normalized size of the lesion in the specific brain region. The normalized size of the lesion is shown in figure 4B in relation to the entire telencephalon, or the targeted lesion area in figure 4C. The success rate was defined as the ratio of pre- to post-surgery success, where a ratio of less than one indicates less success, and above one represents more success. The results showed that there was no clear trend between lesion size and the success rate. Hence, there was no residual dependency on lesion size and most of the effect could be ascribed to the presence of the lesion.

## 4. Discussion

Our study focused on the functional neuroanatomy in teleost fish in relation to hydrostatic pressure and its use during navigation. For this purpose, we combined behavioural experiments and lesion studies in both the LP and MP. Four groups of fish engaged in a vertical task where depth sensing was essential for successful performance. We had four groups: control (no surgery), sham surgery, MP and LP lesion groups. We tested each group’s ability to retain the navigational skill they acquired prior to the lesion. The aim was to establish whether these regions of the telencephalon are related to hydrostatic pressure sensing the navigation along the vertical dimension.

We found that both the LP and MP groups evidenced a decline in their success rate to chance after surgery. In contrast, the control and sham groups maintained a similar success rate as in pre-surgery. This is an indication that these two regions are critical for the processing of hydrostatic pressure information. However, it is clear that additional lesion groups should be tested for the broader picture of hydrostatic pressure coding to be fully provided. Such groups should include different areas of the telencephalon, such as the dorsal and central regions. Also, it would be interesting to include a lesion group to a presumably unrelated brain region as an additional, this time lesioned, sham group.

To the best of our knowledge, this is the first report exploring how hydrostatic pressure may be processed in the fish brain. The results are in line with previous works showing that both the LP and MP are involved in the processing of landmarks and other cues during navigation in the goldfish [16,17,19]. Studies have indicated that neurons that represent the locations and kinematics of the fish are found in the lateral and central parts of the pallium [13,14]. Our results corroborate these findings and suggest that hydrostatic information is processed along with other navigation-related information in these regions.

In addition to the functional neuroanatomy findings, this is the first analysis of hydrostatic pressure in goldfish. The findings are consistent with a previous study on Mexican tetra [9]. Given that these two species belong to different orders, this may be an indication that this capacity could be widespread among different fish, however, these results are not enough for a certain conclusion about other more distant classes of teleost.

### 4.1. Limitations of the current study

Our goal was to determine which regions in the fish telencephalon are involved in depth perception. However, there may be other regions involved in this process both in the telencephalon and outside of this region. This is particularly important since little is known about sensory input to the teleost telencephalon.

In addition to the analysis of the overall success rate after lesion surgery, we controlled for correlations between performance and lesion size. It was expected that the success rate would decrease with the increase in lesion size. We found that this correlation was low, with high variability possibly due to the noisy lesion measurement in all studies.

To address these issues, further studies are needed. Currently, there is a lack of consensus about the division of regions of the goldfish telencephalon [21,22], so the field needs to converge to such a consensus. Then, together with techniques to lesion specific regions, it will allow high-precision targeting of lesions in specific areas. Furthermore, molecular techniques also allow a transient silencing of brain regions and provide insights into the functional neuroanatomy of animals in general and teleost in particular. Part of this effort materialized in the work of Tibi *et al.* [23] who mapped the goldfish telencephalon and found possible homologues to subregions of the mouse hippocampal formation, which are located in the LP region. However, these findings were based on only a few molecular markers. To conclude, it is premature to associate any goldfish telencephalon regions with specific regions in the mammalian brain. Moreover, the concept of the stereotyped one-to-one homology between teleost and mammalian pallial subregions has been increasingly questioned [24]. In particular, depth sensing likely existed in the common ancestor of vertebrates, and was lost (or at least became less important) in terrestrial vertebrates like mammals. To conclude, the current study suggests that the lateral part of the teleost pallium, known to be important for spatial memory, also plays a role in depth sensation. This indicates that ‘the hippocampal function’ identified originally in mammals may not be the only function of the homologous region in aquatic vertebrates.

### 4.2. Broader lesion effects

A successful trial was defined as reaching the correct target location on the first attempt within the time limit. Hence, there were two ways in which a fish could fail. First, to reach for the incorrect basket, and second, to not attempt any basket in the allotted time. While both options occurred in each of the lesion groups, ignoring the baskets altogether was the more prominent option. This raises the question of whether the lesions had a broader effect on the fish other than just diminishing its hydrostatic pressure discrimination capacity.

Previous horizontal navigation lesion studies were conducted using the same lesioning techniques and methodology [10,16]. In these studies, fish that were unsuccessful post-lesion surgery still participated in the tasks given but with reduced success rates. In the current study, we see that the fish are active within the vertical water tank, just uncooperative with the task. While we cannot guarantee that the lesions did not have any additional effects that made the fish unengaged with the task, looking back at other studies shows us that if this was indeed the case, it could have been connected specifically to the exploration of the vertical axis as it did not manifest in the horizontal tasks.

### 4.3. Sensory modality

One critical component of hydrostatic perception remains determining which peripheral organ is responsible for this sensory information. Theoretically, pressure changes in fish can be measured by differences in the volume of the swim bladder, an internal gas-filled organ [25]. This organ is used by the teleost to reduce the cost of vertical locomotion by adjusting the fish’s overall density to be similar to the aquatic environment. This is achieved by continuous adjustments of the amount of gas in the swim bladder by a gland located on the swim bladder wall. This adjustment presents an inherent difficulty since the pressure difference between the swim bladder walls does not monotonically depend on depth. However, theoretical calculations have indicated that this difficulty can be overcome [3,26]. Thus, information from sensors on the swim bladder may innervate upstream information-processing regions in terms of the pressure-related sensing. While the exact location in the central nervous system for swim bladder reflexes is unknown, the diencephalic secretory centre has been put forward as a possibility [27] and may warrant a similar lesion study.

Overall, these results contribute to the growing body of knowledge on the neural basis of teleost fish navigation. They provide the first block of data on the perception of hydrostatic pressure, a critical component in the natural environment of fish in general. Further studies are needed to achieve a greater understanding of this important capacity.

## Data Availability

Supplementary material is available online [28].
